# Impacts of public health and social measures on COVID-19 in Europe: a review and modified Delphi technique

**DOI:** 10.3389/fpubh.2023.1226922

**Published:** 2023-08-31

**Authors:** Marília Silva Paulo, Mariana Peyroteo, Mélanie R. Maia, Cara Pries, Claudia Habl, Luís Velez Lapão

**Affiliations:** ^1^CHRC, NOVA Medical School, Faculdade de Ciências Médicas, NMS, FCM, Universidade Nova de Lisboa, Lisbon, Portugal; ^2^Institute of Public Health, College of Medicine and Health Sciences, United Arab Emirates University, Al Ain, United Arab Emirates; ^3^UNIDEMI, Department of Mechanical and Industrial Engineering, NOVA School of Science and Technology, Universidade NOVA de Lisboa, Caparica, Portugal; ^4^LASI, Laboratório Associado de Sistemas Inteligentes, Guimarães, Portugal; ^5^Gesundheit Österreich GmbH (Austrian National Public Health Institute), Vienna, Austria; ^6^WHO Collaborating Center for Health Workforce Policy and Planning, Instituto de Higiene e Medicina Tropical, Universidade NOVA de Lisboa, Lisbon, Portugal

**Keywords:** COVID-19, health policy, public health and social measures, non-pharmaceutical interventions, modified Delphi expert consensus process

## Abstract

**Introduction:**

The emergence of the COVID-19 pandemic in early 2020 led countries to implement a set of public health and social measures (PHSMs) attempting to contain the spread of the SARS-CoV-2 virus. This study aims to review the existing literature regarding key results of the PHSMs that were implemented, and to identify the PHSMs considered to have most impacted the epidemiological curve of COVID-19 over the last years during different stages of the pandemic.

**Methods:**

The PHSM under study were selected from the Oxford COVID-19 Government Response Tracker (OxCGRT), supplemented by topics presented during the Rapid Exchange Forum (REF) meetings in the scope of the Population Health Information Research Infrastructure (PHIRI) project (H2020). The evidence- based review was conducted using Preferred Reporting Items for Systematic Reviews and Meta-Analysis (PRISMA) guidelines to identify which reviews have already been published about each PHSMs and their results. In addition, two modified Delphi panel surveys were conducted among subject matter experts from 30 European countries to uphold the results found.

**Results:**

There were 3,212 studies retrieved from PubMed, 162 full texts assessed for eligibility and 35 included in this PHSMs summary. The measures with clearest evidence on their positive impact from the evidence-based review include social distancing, hygiene measures, mask measures and testing policies. From the modified Delphi panel, the PHSMs considered most significant in the four periods analyzed were case isolation at home, face coverings, testing policy, and social distancing, respectively.

**Discussion:**

The evidence found has significant implications for both researchers and policymakers. The study of PHSMs’ impact on COVID-19 illustrates lessons learned for future pan- and epidemics, serving as a contribution to the health systems resilience discussion. These lessons, drawn from both the available scientific evidence and the perspectives of relevant subject matter experts, should also be considered in educational and preparedness programs and activities in the public health space.

## 1. Introduction

Since the emergence of the COVID-19 pandemic in early 2020, countries all over the world have selected and implemented several Public Health and Social Measures (PHSMs) in the process of trying to contain the spread of the SARS-CoV-2 virus. These clusters of PHSMs have significantly impacted the population and their application has been questioned in the political, social, and economic dimension.

In the present paper, instead of non-pharmaceutical interventions (NPIs), the concept of PHSMs is used as suggested by the World Health Organization (WHO), due to its clear and inclusive characteristics that describe public health and social interventions as “measures or actions by individuals, institutions, communities, local and national governments and international bodies to slow or stop the spread of an infectious disease, such as COVID-19” (1).

Many health-related policy measures that were applied to different degrees across the world, in combination or individually, can be considered PHSMs. These are usually a set of public health and social tools that have proved effective in limiting the spread and reducing the incidence and prevalence of infections during previous epi- or pandemic outbreaks, such as influenza A H1N1 (2).

During the early stages of the SARS-CoV-2 pandemic in March–May 2020, health systems preparedness, resilience, and capacity response in terms of allocating healthcare workers to combat shortages were considered foremost priorities and received a considerable amount of political attention (3). Still, after 3 years, these issues appear not to have found reflection in mid- to long-term policies, making it imperative to question the actual preparedness of health systems for future crisis events. Several stakeholders have developed interactive maps, dashboards, and catalogs summarizing PHSMs applied per country over time (4, 5). International agencies and universities, specifically the European Center for Disease Control (ECDC) and the University of Oxford, have created interactive maps displaying the epidemiological evolution of the pandemic and the PHSMs applied by countries with the aim of informing the public. Specific platforms were also built to communicate the changeable status of specific PHSMs. As an example, Re-Open EU was launched to provide up-to-date information on applicable travel and health measures in the European Union (EU) and the Schengen Associated countries (6).

At the same time, collaborative European projects conducted by Member State institutions and supported by EU programs, have arisen to address the need for a distributed research infrastructure on population health information as well as the need for rapid *ad-hoc* exchanges of information on health research and policy between countries during the pandemic. The Population Health Information Research Infrastructure (PHIRI) project maintains a continuously updated database of currently applicable PHSMs pertaining to COVID-19 in the participating countries, as well as of relevant research infrastructures, national health information sources, and training resources via the public Health Information Portal (7). PHIRI also conducts bi-weekly Rapid Exchange Forum (REF) gatherings between national project members and expert stakeholders from supra-national institutions to discuss specific urgent topics (8). Questions and topics discussed during each REF meeting are based on countries’ public health institutions’ requests, reflecting their most pressing needs for *ad-hoc* Pan-European information exchange on national population health developments, policies and research. To date, the REF meetings have addressed such questions particularly in the scope of COVID-19 and its impact on different dimensions of population health and health systems.

Strengthening and improving the resilience of health services requires proper and focused policymaking. Therefore, it is essential to understand how health-related policies and measures helped to contain the spread of the SARS-CoV-2 virus and which combinations of measures may have had the highest positive impact.

This paper aims to review the existing literature regarding the key effects of selected PHSMs, substantiate the findings through surveys with subject matter experts, and identify the PHSMs considered to have most impacted the epidemiological curve of COVID-19 over the last years during four different periods of the pandemic.

## 2. Methods

To continuously map COVID-19 health-related policies, indicators and impact measures considered to influence the pandemic’s epidemiological curve were identified. Analysis of those indicators selected on the basis of relevant scientific literature and countries’ requests via the PHIRI REF mechanism, were organized by PHSMs (indicator) in an evidence-based review.

### 2.1. Selection of indicators

Most of the indicators were selected from the Oxford COVID-19 Government Response Tracker (OxCGRT) which was developed by the Blavatnik School of Government, University of Oxford. The OxCGRT collected publicly available information on 24 indicators of government response to the pandemic, three of which were retired before the end of the Tracker’s active collection and publication period. The indicators included containment and closure policies such as school closures and restrictions in movement, economic policies, health system policies such as the COVID-19 testing regime, emergency investments into healthcare, and vaccination policies, among others. The OxCGRT collected and published real-time updates on different policy responses from 1st January 2020 to 31st December 2022, covering more than 180 countries, coded into multiple indicators (9, 10). For this study, 13 indicators were selected from among the OxCGRT indicators, particularly its categories C (containment and closure policies) and H (health systems policies) (11). Two of the selected indicators were modified based on topics presented in and priorities identified during the REF meetings in the scope of the PHIRI project (i.e., closure of kindergartens was added to the school closures indicator; aspects of several indicators within the OxCGRT category V, vaccination policies, were collated under one indicator). Furthermore, six indicators not individually tracked in scope of the OxCGRT were added to the selected indicators through the REF meeting mechanism (i.e., Closure of non-essential shops, gastronomy and cultural venues; Access restriction to shops, gastronomy and cultural venues; Social distancing; General hygiene measures; Voluntary quarantine by contact persons; Case isolation at home), arriving in total at 19 indicators, respectively PHSMs, under study. The 19 PHSMs under study were grouped into five clusters: access measures; distancing measures; movement restrictions; test, trace, vaccinate; communication measures (please see Table 1).

**Table 1:** Reviews reporting on the selected public health and social measures found.

Clusters	Non-pharmaceutical interventions	References
Access measures	Closure of schools and kindergartens	(12–24)
	Workplace closure	(21, 23)
	Closure of non-essential shops, gastronomy, and cultural events	(21, 24)
	Access restrictions to shops, gastronomy, and cultural events	No studies assessing this PHSM
	Cancelation of public events	(23, 25)
Distancing measures	Restriction on public gatherings	(23, 25)
	Social distancing	(14, 16, 20–22, 24, 26–30)
	Hygiene measures	(16, 24, 31)
	Face coverings (all types)	(22, 24, 26, 31–34)
	Voluntary quarantine by contacts	(22, 35)
Movement restrictions	Case isolation at home	(20, 22, 23, 27, 28, 35, 36)
	Public transports closures	(21)
	Stay-at-home campaign	(14, 15, 27, 29, 36)
	Restrictions international movement	(15, 25, 28, 29, 37–39)
	International travel control	(22, 28, 29, 37, 40)
Test, trace, vaccinate	Testing policy (anyone with symptoms)	(35, 36, 41, 42)
	Contact tracing	(13, 15, 16, 19, 28, 35, 36, 42–46)
	Vaccination policy (all vulnerable groups)	No studies assessing this PHSM
Communication measures	Public information campaigns	No studies assessing this PHSM

### 2.2. Review of evidence research – reviews

This review of reviews aims to provide a summary of different reviews on PHSMs to answer our research question “Which PHSMs can be considered to have most impacted the epidemiological curve of COVID-19 from 2020 to 2022?.” This type of review was introduced in healthcare due to the high number of health interventions and clinical studies that exist, and the Cochrane group has described it as an Overview of Reviews (47). Review of reviews have a similar structure to systematic reviews but include reviews rather than primary studies. Therefore, we have used the PRISMA statement guidelines to report the review (48).

#### 2.2.1. Eligibility criteria

Studies had to meet the following criteria: any type of review presenting structured methods and evidence of the impact of the selected PHSMs on reducing the epidemiologic curve of COVID-19. Exclusion criteria were a focus on the impact of PHSMs on topics outside of general population health (e.g., mental health) and on diseases other than COVID-19.

#### 2.2.2. Information sources and search string

We developed a search string on PubMed MEDLINE using a combination of terms relating to the pandemic, the development of this search string focusing on the exposure (COVID-19) was very comprehensive: “covid 19”[Title/Abstract] OR “covid 19”[Title/Abstract] OR “covid 2019”[Title/Abstract] OR “COVID19”[Title/Abstract] OR “COVI-19”[Title/Abstract] OR “covid 2019”[Title/Abstract] OR “2019 ncov”[Title/Abstract] OR “2019 ncov”[Title/Abstract] OR “coronavirus disease 19”[Title/Abstract] OR “coronavirus disease 19”[Title/Abstract] OR “coronavirus disease 2019”[Title/Abstract] OR “coronavirus disease 2019”[Title/Abstract] OR “2019 novel Coronavirus”[Title/Abstract] OR “COVID19”[Title/Abstract] OR “coronavirus disease 2019”[Title/Abstract] OR “2019-coronavirus”[Title/Abstract] OR “coronavirus-2019”[Title/Abstract] OR “2019-coronavirus”[Title/Abstract] OR “coronavirus-2019”[All Fields] OR “SARS Coronavirus 2”[Title/Abstract] OR “sars cov 2”[Title/Abstract] OR “sars cov 2”[Title/Abstract] OR “CoV-2”[Title/Abstract] OR “COVID-2”[All Fields] OR “Wuhan coronavirus”[Title/Abstract] OR “covid 19”[MeSH Terms:noexp]. Then it was combined with all the indicators selected using the Title/Abstract term in the query box. No filters apart from limiting the search to the type of study eligible and to studies published in the English language were applied.

#### 2.2.3. Selection process

All the citations retrieved from PubMed were uploaded into Covidence – Better systematic review management (49), which automatically removes duplicates and is ready to provide a PRISMA flowchart. A thorough screening of titles and abstracts followed by full-text screening was performed by four reviewers. Each methodological step was conducted by two independent researchers on Covidence to ensure their eligibility to be used to produce a narrative of the key results per PHMS.

#### 2.2.4. Data items

Data was extracted using a prepared and specific sheet focusing on the type of scientific paper, the PHSMs reported, the type of review (type of study), the period where the study was conducted, the type of studies included by study design, and the number of studies included, the key results and recommendations if any. The data extraction items were tested in an excel sheet by two reviewers and then inserted into Covidence to allow double extraction. Each reviewer was blinded during the data extraction stage and a final data sheet was created highlighting any conflicts between the two extractors. The conflicts were all solved by the same author to ensure consistency. This last data extraction sheet was downloaded, and results were summarized by cluster of PHSM.

#### 2.2.5. Study risk of bias assessment

Risk of bias in the studies included was assessed by a tool developed by Whiting and colleagues to assess the risk of bias in systematic reviews – ROBIS (50). This tool focuses on assessing four domains, comprising the adequate objectives and eligible criteria, the search and databases used, the study characteristics provided, and the synthesis of results, plus, an overall risk domain.

### 2.3. Modified Delphi technique

The modified Delphi technique sought to elucidate the most significant PHSMs applied in European countries during different periods of the pandemic in 2020-2021 by studying the perception of relevant subject matter experts from the PHIRI project network. The Delphi techniques are still evolving, but they can be defined as a complex method to structure group communication processes to reach a consensus, based on collected experts’ judgments (51).

#### 2.3.1. First and second-round surveys

A two-round survey was designed and distributed to subject matter experts from all 30 countries participating in the PHIRI project. In the scope of the survey, subject matter experts were defined as members of the PHIRI project consortium and network who have been actively engaged in COVID-19 responses throughout the pandemic in the scope of research, design of policy, or advice to policymakers. This includes experts from side of national authorities such as Ministries of Health, national public health institutes, or other relevant government agencies and research institutions. In the first stage of the survey, the subject matter experts were asked to select, by way of multiple-choice questions from a list of initially 19 included measures, the three PHSMs which they considered had most impacted the epidemiological curve of COVID-19 during each period of the pandemic (March–May 2020, September 2020–February 2021, March–May 2021 and October–December 2021). After receiving the responses from the first round, analysis was performed. All the PHSMs that were selected among the most impactful measures by the majority of respondents of the first survey were taken forward for inclusion in the second round. The second survey was created from the resulting list of PHSMs per surveyed period, and again distributed to all members of the PHIRI project network. In this second survey, subject matter experts from each PHIRI member country were asked to rank the remaining PHSMs in order of their relative importance to decreasing or controlling the epidemiological curve of COVID-19 in each period of the pandemic, to arrive at a ranking of PHSMs deemed most impactful overall in each period by participants of the Delphi Panel.

## 3. Results

### 3.1. Evidence-based review

**Figure 1:**
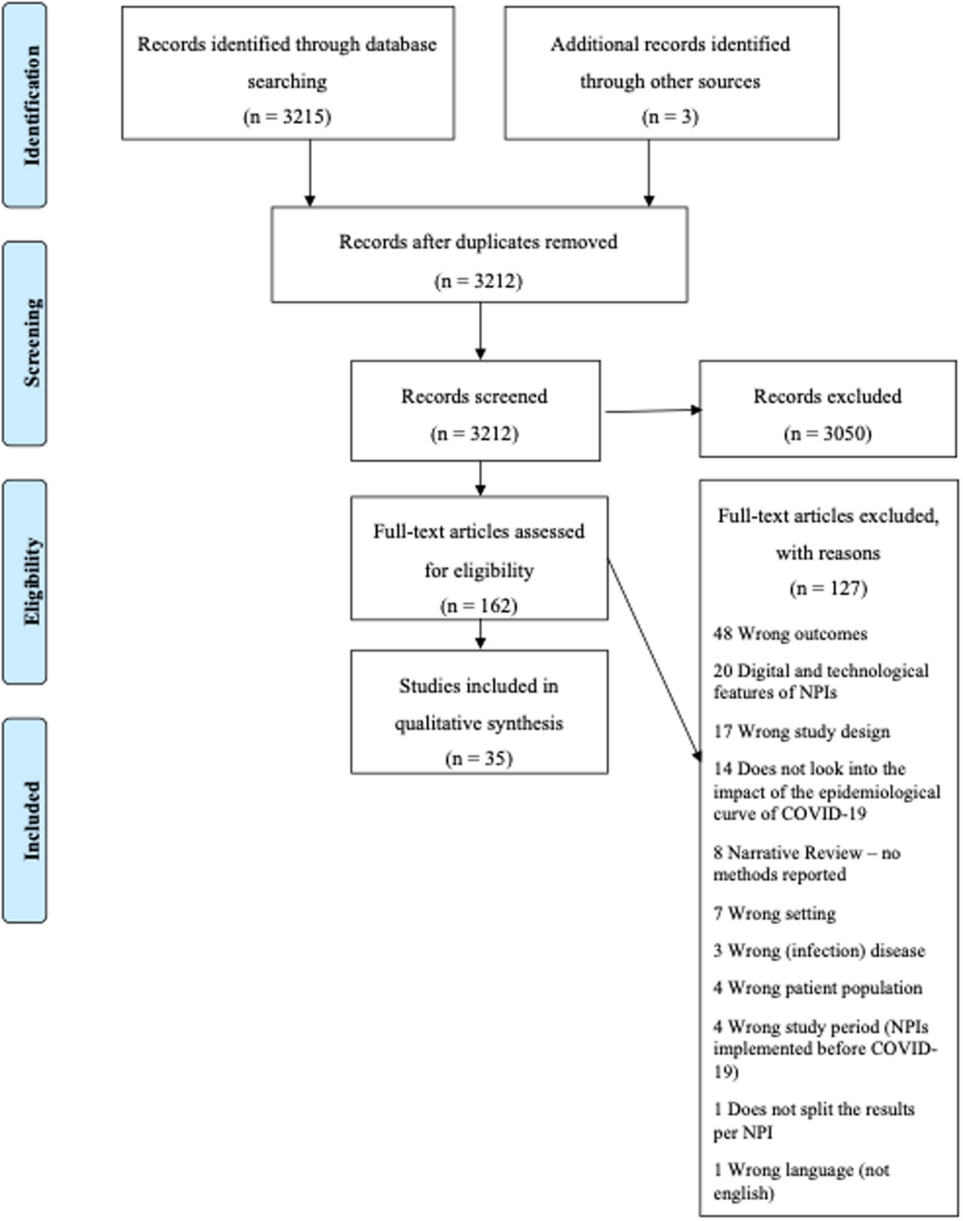
PRISMA-flow chart.

The search performed identified 3,212 citations. After duplicate removal, 3,050 were considered ineligible at the titles and abstract screening stage, 162 were screened for full-text and 35 were included (Figure 1).

Of the 35 studies included, 16 were systematic reviews, 7 were systematic reviews and meta-analyses, 8 were rapid reviews, three were scoping reviews, and one was an evidence-based review. The number of papers included in these studies varied from 9 to 90 and all reviews addressed multiple countries. Most of the studies, 17 reviews, examined more than one PHSM in the same paper. The most studied PHSM was social distancing (Table 2).

**Table 2:** Table of characteristics of the studies included.

Study ID	Type of review	Databases included	Start date	End date	Type of studies included	Number of studies included	Public health and social measure
Abboah-Offei 2021 (32)	Rapid review	CINAHL^1^, Embase, Global Health, MEDLINE, PsyInfo	Starting of the pandemic	Jun-20	RCTs^2^; Retrospective cohort; Case–control; Cross-sectional; Descriptive, Observational; Reviews; systematic reviews, Protocols, opinions, discussion papers	58	Face coverings
Ayouni 2021 (22)	Systematic review	CINAHL^1^, Cochrane Library, Embase, Gray.net, MEDLINE, Preprint databases, ProQuest, PubMed, Scopus, Web of Sciences, WHO^3^ website	Jan-20	Mar-21	RCTs^2^; Retrospective cohort; Case–control; Descriptive, Observational	18	Closure of schools, Contact tracing, Face coverings, Social distancing, International travel control, Testing policy
Bou-Karroum 2021 (37)	Systematic review	Cochrane Central Register of Controlled Trial, Embase, MEDLINE, PubMed	Jan-20	Dec-20	RCTs^2^; Retrospective cohort; Case–control; Descriptive, Observational	69	Restrictions international movement; International travel control
Caristia 2020 (39)	Rapid review	WHO COVID-19 Database	Not reported	5, July 2020	Descriptive, Observational	19	Stay-at-home campaign
Chu 2020 (26)	Systematic review and meta-analysis	CINAHL^1^, Embase, PubMed, Ovid, WHO Global Index Medicus	Not reported	May-20	Descriptive, Observational	44	Face coverings, Social distancing
Chung 2021 (36)	Systematic review	Cochrane Library, JSTOR, MEDLINE, Scopus	Jun-20	January 2021	RCTs^2^; Descriptive, Observational	118	Contact tracing, Social distancing, Voluntary quarantine by contacts, Restrictions on international movement, Testing policy
Ford 2021 (34)	Systematic review	CINAHL^1^, Embase, PubMed, WHO COVID-19 Research Database	Jan-20	Mar-21	Reviews	21	Face coverings
Girum 2020 (35)	Systematic review	CINAHL^1^, Clinical Trials registries, Embase, Global Health Database, Google Scholar, PubMed	Not reported	Jun-20	RCTs^2^; Descriptive, Observational	22	Case isolation at home, Contact-tracing, Voluntary quarantine by contacts, Testing policy
Girum 2021 (29)	Systematic review	CINAHL^1^, MEDLINE, PsyInfo, Scopus, Web of Sciences	Jan-20	Jun-20	Descriptive, Observational	25	International travel control, Restrictions international movement, Stay-at-home campaign, Social distancing
Grépin 2021 (40)	Systematic review	BioRxiv, MedRxiv, PubMed	Not reported	Jun-20	Descriptive, Observational	29	International travel control
Hossain 2022 (46)	Systematic review	Cochrane Library, Embase, PubMed	Not reported	Nov-21	RCTs^2^; Descriptive, Observational	47	Contact tracing
Iezadi 2021 (14)	Systematic review and meta-analysis	Multiple COVID-19 Research DBs	late December 2019	February, 12,021	Retrospective cohort; Cross-sectional	35	Stay-at-home campaign, Social distancing
Irfan 2021 (15)	Systematic review and meta-analysis	Cochrane COVID-19 Study Register, DC COVID-19 Research Articles Downloadable Database for BioRxiv, Embase, MEDLINE, MedRxiv, SSRN preprints, WHO COVID-19 Global literature on coronavirus disease	Dec-19	Apr-21	Descriptive, Observational	90	Closure of school and kindergartens,
Jenniskens 2021 (44)	Rapid review	BioRxiv, Embase (Ovid), MEDLINE, MedRvix, PubMed	Not reported	28-Oct-20	RCTs^2^; Retrospective cohort; Case–control; Cross-sectional; Descriptive, Observational	17	Contact tracing
Khosravizadeh 2022 (30)	Systematic review	Google Scholar, Irandoc, Magiran, PubMed, Science Direct, SID, Scopus and Web of Sciences	2019	Mar-21	Reviews	13	Social distancing
Krishnaratne 2020 (17)	Scoping review	CINAHL^1^, PubMed, Scopus	08-Oct-20	Not reported	Case–control; Descriptive, Observational	42	Closure of schools and kindergartens
Krishnaratne 2022 (16)	Rapid review	Cochrane Central Register of Controlled Trials, Cochrane COVID-19 Study Register, Embase, MEDLINE, WHO COVID-19 Global literature on coronavirus disease	09-Dec-20	Not reported	Reviews	38	Closure of schools and kindergartens
Mazza 2021 (43)	Systematic review	Cochrane Library, Embase, PubMed, Web of Sciences	Nov-19	Apr-21	Case–control; Cross-sectional; Descriptive, Observational	10	Contact tracing
Mbwogge 2021 (42)	Systematic review	Google Scholar, Mendeley, PubMed, Science Direct	Sep-20	Dec-20	Descriptive, Observational	35	Contact tracing, Testing policy
Mendez-Brito 2021 (23)	Systematic review	Cochrane Library, Embase, MedRxiv, PubMed	January 1 2020	March 4 2021	RCTs^2^; Descriptive, Observational	34	Closure of schools and kindergartens, Restriction on public gatherings, Social distancing, Public transports closure, Stay-at-home campaign, International travel control
Nussbaumer-Streit 2020 (38)	Rapid review	CINAHL^1^, Embase, Ovid MEDLINE, PubMed, WHO Global Index Medicus	Not reported	23-Jun-20	Retrospective cohort; Case–control; Descriptive, Observational	51	Restrictions international movement
Patiño-Lugo 2020 (28)	Systematic review	Embase, MEDLINE	01-Jan-20	25-Mar-20	Descriptive, Observational	9 + 128 webpages	Case isolation at home, Contact tracing, Restrictions international movement, Social distancing
Rizvi 2021 (20)	Systematic review and meta-analysis	BioRxiv, Google Scholar, MEDLINE, MedRxiv, PubMed, Welcome Open Research	Not reported	27-Mar-20	Descriptive, Observational	28	Closure of schools, Social distancing
Shah 2020 (27)	Systematic review	Embase, Google Scholar, MEDLINE, Scopus, WHO database	Dec-19	15-Jun-20	Descriptive, Observational	13	Case isolation at home
Sun 2022 (21)	Scoping review	AMED, Embase, Global Health, MEDLINE, PsyInfo, Ovid Nursing Database, Social Work Abstracts	Not reported	30-Sep-20	Cross-sectional; Descriptive, Observational; Reviews	41	Closure of schools and kindergartens, Closure of non-essential business, Social distancing, Workplace closure
Talic 2021 (24)	Systematic review and meta-analysis	Embase, MEDLINE, MedRxiv	Not reported	07-Jun-21	RCTs^2^; Descriptive, Observational	72	Closure of schools and kindergartens, Face coverings, Hygiene measures, Stay-at-home campaign, Social distancing, Workplace closure
ThomasCraig 2021 (45)	Systematic review	MedRxiv, PubMed	Jan-20	24-Jul-20	Descriptive, Observational	24	Contact tracing
Tran 2021 (33)	Scoping review	Assia, ClinicalTrial.gov, Cochrane, Embase, Google Scholar, PubMed, System for Information on Grey Literature in Europe (SIGLE), Scopus, Web of Sciences	20-Feb-20	09-Jul-20	RCTs^2^	27	Face coverings
Viner 2020 (18)	Rapid review	China National Knowledge Infrastructure (CNKI) Database, Google Scholar, Latin American and Caribbean Health Sciences Literature (LILACS), WanFang Database	March 9 2020	March 19 2020		16	Closure of schools and kindergartens
Viner 2021 (19)	Systematic review and meta-analysis	MedRxiv, PubMed	Not reported	Not reported	Descriptive, Observational	16	Closure of schools and kindergartens, Contact tracing
Viner 2022 (13)	Systematic review and meta-analysis	COVID-19 Living Evidence database, Europe PMC, MedRxiv, PubMed	Jan-20	Jul-21		43	Closure of schools and kindergartens, Contact tracing
Viswanathan 2020 (41)	Rapid review	CDC COVID-19 Research Articles Downloadable Database, CENTRAL, Cochrane COVID-19 Study Register, Covid-Analytics, Embase, MEDLINE, Models of Infectious Disease Agent Study, PubMed	Not reported	26-May-20	Retrospective cohort; Descriptive, Observational	22	Testing policy
Walsh 2021 (12)	Systematic review	CINAHL^1^, ERIC, Google, PubMed, Scopus, the Australian Education Index, the British Education Index, Web of Sciences, WHO Global COVID-19 Research Database	2020	07-Jan-21	Descriptive, Observational	40	Closure of schools and kindergartens
Walsh 2022 (25)	Rapid review	Cochrane, Embase, Europe PMC, Google, MEDLINE, Web of Sciences	01-Jan-20	03-Jun-21	Descriptive, Observational	11	Cancelation of public events, Restriction on public gatherings
Yuen 2021 (31)	Evidence-based review	MedRxiv, PubMed, Science Direct	Not reported	Not reported	Reviews	54	Face coverings, Hygiene measures

^1^Cumulative Index to Nursing and Allied Health Literature. ^2^Randomized Control Trial. ^3^World Health Organization.

Of the studies included, 23 (64%) had performed risk of bias assessment and another 23 (64%) had performed quality of evidence assessment of the studies included in their analysis (either quantitative or qualitative). From the risk of bias assessment performed, we assessed 33 studies as overall low risk of bias and two studies as unclear risk. The domains that contained more concerns were related to the search and the selection of databases (“*Did the search include all appropriate range of databases for published and unpublished studies? Were methods additional to database search used to identify relevant reports?*”) and to the sufficient studies characteristics available (“*Were sufficient study characteristics available for both review authors and readers to be able to interpret the results?*”). Please see the Supplementary material for a detailed risk of bias assessment.

The results are organized by PHSM, providing a summary of evidence on each measure. Table 1 shows an overview of the studies included for each PHSM.

#### 3.1.1. Access measures

Thirteen studies assessed the impact of **school and kindergarten closure** on the transmission and incidence of SARS-CoV-2 (12–24). Six studies recommend the implementation of this PHSM with caution (12, 16, 20, 22–24) and four showed inconclusive results because it must be considered that the studies evaluated a set of combined measures (e.g., social distancing) and not this measure alone (13, 17, 19, 21). Nevertheless, eight studies showed evidence of positive impact on the epidemiological curve when the measure was implemented in periods of low incidence of SARS-CoV-2. One study assessed the impact of **workplace closures**, whereby it was possible to conclude that implementing this measure had a moderate impact on reducing transmission (21). As for the **closure of non-essential shops, gastronomy and cultural events**, two studies reported a significant reduction (between 12 and 29%) in SARS-CoV-2 transmission associated with this measure (21, 24). In the studies reviewed, there was no assessment of the impact of **canceling public events** on reducing the epidemiological curve of COVID-19.

#### 3.1.2. Distancing measures

The most frequently implemented measures during **public gatherings** were providing hand disinfectant, wearing face masks, ensuring adequate ventilation, symptoms screening (i.e., temperature, symptom, travel, or close contact screening) and contact tracing. One study presented evidence that implementing a range of PHSMs can reduce the risk of SARS-CoV-2 transmission at mass gatherings (25); however, this risk is unlikely to be eliminated. All studies adopted a layered mitigation approach involving multiple public health measures; therefore, the effectiveness of any single measure under the umbrella of restrictions on public gatherings could not be determined. Some studies considered physical distancing, quarantine and contact tracing as part of the results analysis of **social distancing** (24, 26). For **hygiene measures**, the interventions studied fell into three broad categories: the main use of hand sanitizers, the use of soap, and those that provided education on hygiene practices only (24, 31). Further recommendations to be applied in specific contexts such as using gloves, gowns, and eye protection, saline nasal washing and gargling were also identified. From the included studies, we can infer that the implementation of hygiene measures had an impact on reducing the incidence of COVID-19. In addition, one study evaluated surface disinfection with chlorine or ethanol-based disinfectant, concluding its effectiveness in reducing SARS-CoV-2 transmission (24). There was consensus among 10 studies that using **face covering** reduces the risk of transmission, incidence, mortality, and hospitalization for COVID-19 (22, 24, 26, 31–34). The impact of not using a mask, using a face mask, surgical or medical masks, and N95 masks were analyzed. The latter may be associated with a greater risk reduction compared to surgical or similar masks according to included studies, particularly when mandatory use of N95 masks is implemented (26, 33). Five included studies demonstrated that **voluntary quarantine by contacts** effectively suppressed transmission of COVID-19 in conjunction with contact tracing. However, one study reports that particular consideration needs to be given to providing appropriate measures for vulnerable populations, as quarantine and screening may not be sufficient to address their needs (27).

#### 3.1.3. Movement restrictions

As with voluntary quarantine of close contacts, **case isolation at home** was shown to be effective in suppressing transmission of COVID-19 (20, 22, 23, 27, 28, 35, 36). However, the effectiveness of this measure has been questioned with increasing evidence of natural immunity and disease resistance.

The two studies that analyzed the impact of the **public transport closure** did not show any significant difference in the progression of the epidemiological curve associated with this measure (21, 23). This may have been due to other measures already implemented during the studied time periods (resulting in low public transport congestion and face masks worn on public transport). One of the studies included an analysis of 12 papers, of which only one found an association between public transport closures and the reproduction number, growth rate, or case-related outcomes of COVID-19 (23).

The **stay-at-home campaign** focused on two complementary measures: lockdown and quarantine. Regarding the impacts of lockdown, all the studies that evaluated stay-at-home or isolation measures reported reductions in transmission, incidence, hospital and ICU admissions, and deaths from SARS-CoV-2. One of the studies reported that a combination of four measures, including restrictions on mass gatherings, school closures, workplace closures, and lockdowns in 32 countries, was associated with decreased incidence of COVID-19 (21). A similar decreasing incidence was observed when public transport closures were added. Quarantine was reviewed in two studies, which concluded that its implementation reports a decrease in the incidence of COVID-19.

The effectiveness of travelers’ quarantine and the need for arrival or exit screening were examined as measures to **restrict international movement**. Included studies show that the effectiveness of traveler quarantine depends on compliance and increases when traveler quarantine is implemented as a mandatory measure. Four studies demonstrate quarantine’s impact, especially for travelers from countries with a high prevalence of SARS-CoV-2 and detecting new cases that were initially negative (22, 36–38). In addition, it was found that reopening borders without travelers’ quarantine measures rapidly increased the number of new cases of COVID-19 (37). Studies also showed that screening travelers allowed for a delay in the next epidemic peak and a reduction in the number of cases compared to earlier peaks. However, in two separate countries lifting travel restrictions did not increase the number of cases when accompanied by other measures of physical distancing and quarantine of travelers. Complementarity of measures, such as quarantine and screening, showed a high impact in reducing the number of cases, mainly when screening was performed before day 14 of quarantine.

The included studies on **international travel control** measures, such as travel restrictions and border closure measures, showed mixed results in the association between border closure and reduction of critical cases or overall mortality from COVID-19 (22, 28, 29, 37, 40). On one hand, reducing transmission between countries appears to be more effective through border closures than screening for symptoms at airports, and is particularly useful in the early phase of an outbreak before the widespread distribution of the disease (36). On the other hand, border restrictions in combination with other measures (quarantine, isolation, social distancing, closure of schools and workplaces, working from home, and restrictions on internal movement) show that together these measures were effective in reducing the number of cases of COVID-19, but could not isolate the impact of international travel control measures. Additionally, one study presented inconclusive results in analyzing domestic and international travel restrictions (38).

#### 3.1.4. Test, trace, and vaccinate

Specific to **testing policies**, most of the studies reviewed conclude that testing policies (along with case isolation, social distancing, and face masks) can effectively control a new outbreak of COVID-19. However, a study assessing the accuracy of screening strategies showed inconclusive results on the usefulness of combined screening, repeated symptom assessment, and rapid laboratory tests (41).

As for **contact tracing**, the nine studies included in this analysis showed the usefulness and benefits that digital tools – Contact Tracking Apps (CTA) with text warning systems – could have in managing an outbreak. Despite promising results on contact tracing policies, two studies highlight that for the results to be effective, these apps need to provide faster feedback on a positive test result and notify close contacts (42, 43). Two other studies mention the need for a high app usage rate (44, 45). Another study mentions that CTA must be combined with other interventions (such as social distancing and random testing) to reduce the epidemiological curve of SARS-CoV-2.

Combining both measures is essential, as one study found that each new case requires an average of 36 individuals to be analyzed, and laboratory testing (within 2 h) can increase the efficiency of this process (35). **Vaccination policy** in terms of the polices and strategies to vaccinate all or certain groups of the population was not addressed in any eligible studies included in this review.

#### 3.1.5. Communication measures

Another PHSM that we investigated were **public information campaigns**, but there were no available studies describing the impact of public health campaigns on the epidemiological curve of COVID-19.

### 3.2. Modified Delphi technique

**Figure 2:**
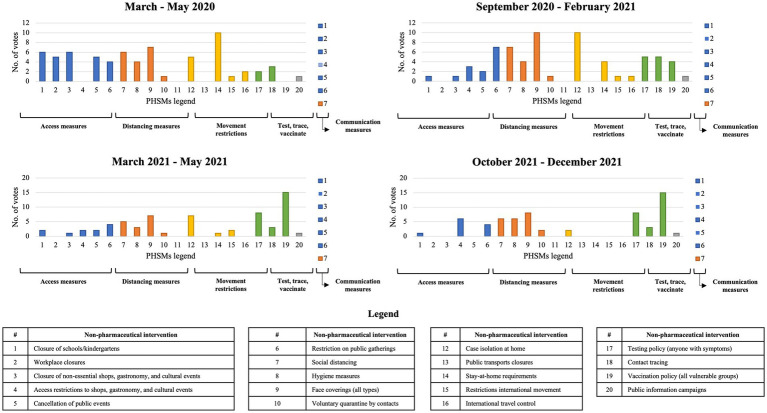
Results from the first Delphi technique round.

The first round of the survey received responses from subject matter experts from Belgium, Bosnia Herzegovina, Croatia, Estonia, Hungary, Italy, Norway, Portugal, Romania, Slovakia, Slovenia and Spain. For the period of March to May 2020, the PHSM considered most impactful among those implemented during this period were Stay-at-home campaigns with a relative majority of 45% survey participants choosing this measure in their responses. For the period of September 2020 to February 2021, the PHSMs deemed most impactful by respondents were “face coverings (of all types)” and “case isolation at home” each chosen by 45% of participants, respectively. For the period of March to May 2021 the PHSM deemed impactful by the highest number of respondents was “vaccination policy” chosen by 68% of participants. Finally, for the period of October to December 2021, the PHSM considered most important overall was again “vaccination policy,” chosen by 68% of participants. Results are presented in Figure 2.

**Figure 3:**
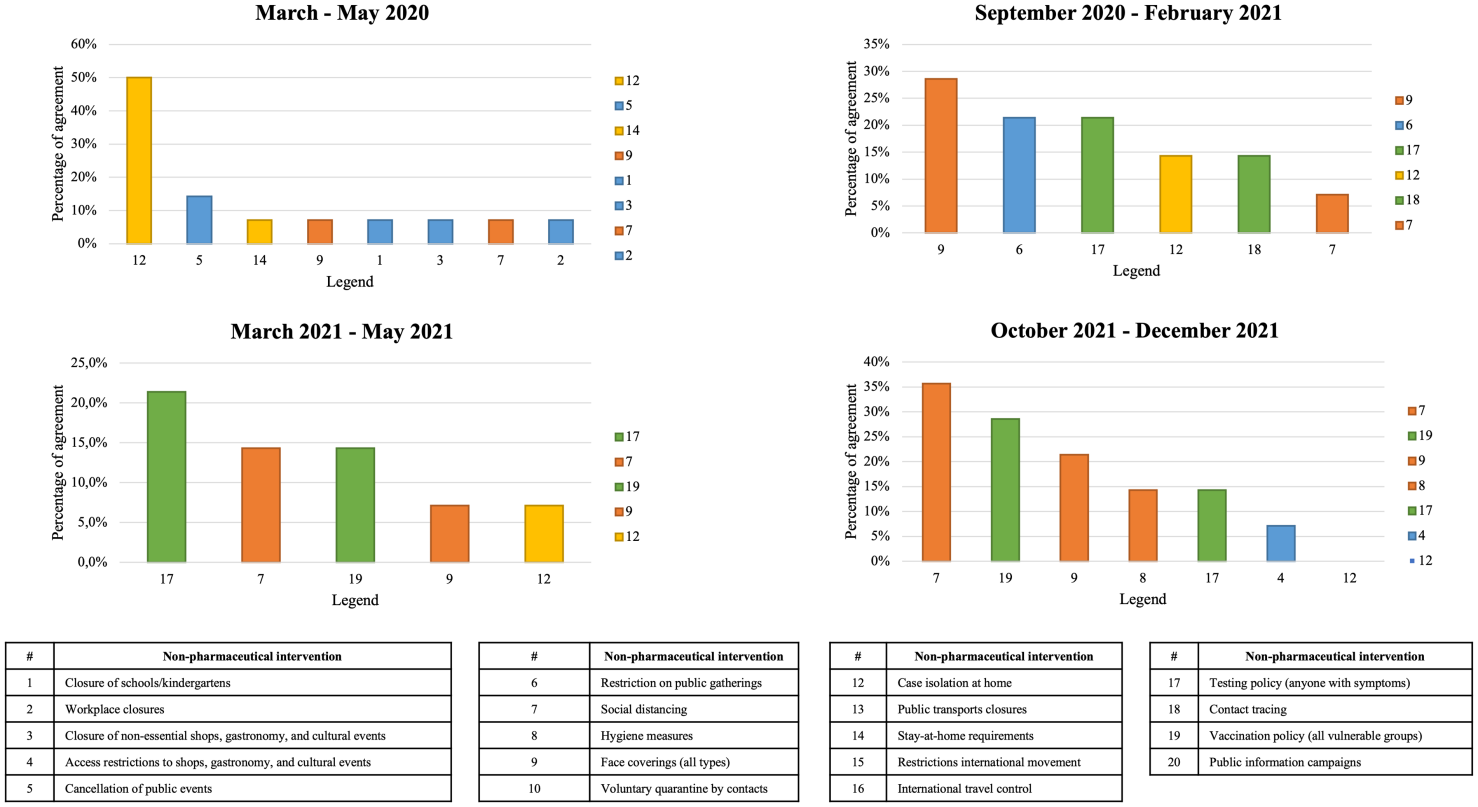
Results from the second modified Delphi technique round.

The second round of the survey received responses from subject matter experts from Albania, Austria, Belgium, Croatia, France, Italy, Norway, Portugal, Slovakia, Spain, and Sweden Public Health Experts. For the period of March to May 2020, the PHSM considered most impactful was “case isolation at home” as chosen by 50% of participants. For the period of September 2020 to February 2021, respondents considered “face coverings (of all types)” as the most relevant measure with 29% of participants choosing this option. For the period of March to May 2021, the PHSM considered most impactful was “testing policy” chosen by 21% of participants, followed by a tie between social distancing and vaccination policy. Finally, for the period of October to December 2021, the PHSM considered most impactful was “social distancing” chosen by 36% of participants, followed by vaccination policy (29%). Results are presented in Figure 3.

## 4. Discussion

### 4.1. Key findings

PHSM are key interventions at the disposal of policymakers in the public health space to address an epi- or pandemic, to limit spread of infectious disease, and to mitigate its impact. From this review, and in the context of COVID-19, one could consider that there are three sets of PHSMs, distinguished by different levels of evidence currently available regarding their impact: one set of PHSMs where there is clear evidence, one set with just moderate evidence, and another with to date still little evidence on their impact.

PHSMs with clear evidence of positive impact from the literature review are closure of non-essential shops, gastronomy and cultural events, hygiene measures, face coverings, voluntary quarantine by contacts, case isolation at home, stay-at-home campaign, restrict internal movement, and testing policies.

PHSMs with a moderate level of evidence, often to be implemented as a combined intervention are workplace closure, restrictions on public gatherings, social distancing, international travel control measures and contact tracing.

PHSMs with little evidence available to date, eventually requiring more studies are closure of schools and kindergartens, cancelation of public events, public transport closure, vaccination policy strategies, and public information campaigns.

Overall, combined interventions have shown to be effective and have a high impact in reducing the transmissibility of the disease, the collapse of health care services, and mortality (28).

Another interesting result lies in the comparison of evidence from the rapid review with the perceptions reported by subject matter experts via the modified Delphi panel technique. During the initial period (from March to May 2020), the PHSM identified as most impactful by subject matter experts was “case isolation at home,” which fits well with the evidence presented in the review and illustrates a strong focus during this period of the pandemic on measures that restricted people from their usual movements and activities.

In the next period (from September 2020 to February 2021), the PHSM identified as most impactful by the experts were “face coverings (of all types),” again aligning with the evidence drawn from the rapid review and representing a shift in focus during this period of the pandemic toward measures that would allow people to leave their homes and be somewhat active while still engaging in preventative measures.

In the following period (from March to May 2021), the PHSM considered most impactful by experts was “testing policy,” followed by a tie between social distancing and vaccination policy, representing a stage of the pandemic where societies were forced to adopt – and continuously adapt – different parallel clusters of measures to keep functioning. It was also the period when COVID-19 vaccination first became available to the population.

In the last surveyed period (from October to December 2021), the PHSM identified as most impactful by experts was “social distancing,” followed by vaccination policy. With most populations already vaccinated at this stage, this result seems to point toward a focus on avoiding infections among vaccinated people, as well as a continued prioritization of raising immunization rates in populations with still low rates of vaccination. Interestingly, the fact that we do not have vaccination policy studies focusing on priorities of the population to be vaccinated, just vulnerable groups vs. all, including children or not, reflected on the need of further studies as the vaccination policies rather just on the vaccines efficacy itself.

The review also identified a set of PHSMs that still require more study to determine their impact during the SARS-CoV-2 pandemic and potential utility in subsequent outbreaks. For instance, the use of contact tracing apps could represent an important tool in the future if their present limitations are addressed, and no eligible studies were identified in scope of this paper which examined the impact of communication measures, such as public information campaigns, pointing to the need for further research. Information technologies are still to make a difference; therefore it represents an area where researchers and policymakers should pay more attention and eventually invest more.

### 4.2. Lessons learned and future implications

The study of PHSMs’ impact on COVID-19 provides significant lessons learned for the expected next pandemic. These lessons should be integrated into both education- and preparedness programs in the public health space, as well as informing the policy decision process in acute phases of a comparable health crisis.

Besides PHIRI, several other European projects and activities have been initiated to address the impact of the COVID-19 pandemic and provide evidence-based support for future decision-making regarding the implementation of effective PHSMs across the region. A closer future collaboration between some of these projects and entities, as well as between the projects and national policymakers across Europe, could both stimulate wider scientific discussion and build additional resilience to fight future health crises armed with learnings from the previous pandemic. Notable European projects and programs in this domain include HERA (European Health Emergency Preparedness and Response Authority), VACCELERATE (Vaccine Infrastructures and Communication for Europe), COVID-19 Social Sciences Research Tracker, RECOVER-E (Rapid European COVID-19 Emergency Research response), CoVaRR-Net (COVID-19 Vaccine-induced Immunity, Variants, and Re-infections Network), and EPIPose (Epidemic Intelligence to Minimize COVID-19 Impacts on European Society, Public Health and the Economy).

When considering the policymakers perspectives, it is clear how the evidence during the COVID-19 pandemic was used – not only taking into consideration its absolute scientific validity but also the social context, whereby the evolution of PHSMs’ relevance follows the pandemic stages and shows a shifting focus on different dimensions of PHSMs over time, alongside new epidemiological developments as well as an evolving societal understanding of and amenability to different measures. Considering the available scientific evidence on PHSMs’ impact, as well as the subjective perspectives of subject matter experts who advise health policy, has allowed the present study to form a nuanced understanding of different measures’ significance over time and to highlight the complex overlapping dimensions of decision-making during a pandemic, which can be taken into account both by national policymakers and the experts engaged in counseling policy during future health crises. For this reason, analyses such as the one conducted in the scope of this paper can form a vital building block in informing health policy processes going forward.

### 4.3. Strengths and limitations

During the COVID-19 pandemic, thousands of scientific papers were published at a very fast pace, particularly in 2020 and 2021, making it difficult to assess and identify the key results to aid decision-making. The choice of performing a review of reviews, therefore, presents as a strength to summarize which PHSMs have most impacted the COVID-19 pandemic in Europe according to available evidence. The second strength to highlight is the use of the PHIRI REF network to engage subject matter experts in the modified Delphi panel, as they represent relevant expertise from many different European Member States and associated countries.

A limitation regarding the rapid review lies in some of the characteristics of available studies that were considered for inclusion. A good proportion of the excluded studies focused exclusively on the impact of PHSMs on outcomes related to anxiety, depression, loneliness and other aspects of mental health, thereby failing to meet the eligibility criteria as defined in the Methods chapter. Many studies conducted at the beginning of the pandemic were solely based on modeling approaches, i.e., intended to support only short-term decision-making. Among observational and modeling studies, we have extracted data only from observational studies. Another limitation of the review lies in the often mutually confounding nature of the PHSMs under study and is reflected in the fact that the available literature often examined a combination of measures from multiple clusters as defined in this paper. However, this circumstance simultaneously presents a strength: While it limits the ability of this study to rank individual PHSMs by the size of their impact on reducing the epidemiological curve of COVID-19 in absolute terms, it allows for observations on the complex interplay of available measures and their effects during different periods of the pandemic that hold meaningful lessons for researchers and policy makers in context of future outbreaks.

In terms of limitations affecting the methodology of the modified Delphi panel survey technique, the subject matter experts’ opinions and views do not represent official data from their countries but personal perception, and this can be seen as a limitation due to the resulting subjectivity. However, in addressing this limitation, we maintain that policy decisions, especially during a fast-moving crisis, are often based on the perception of relevant subject matter experts and the policymakers they advise. Studying the subjective perception of relevant experts in the field, therefore, provides a significant complement and substantiation – another piece of the evidence puzzle – to the results of the literature review.

## 5. Conclusion

The review identified one set of PHSMs with clear evidence of their positive impact – including closure of non-essential shops, gastronomy and cultural events, hygiene measures, face coverings, voluntary quarantine by contacts, case isolation at home, Stay-at-home campaign, restrict internal movement and testing policies – and another set of PHSMs with moderate available evidence, including workplace closure, restrictions on public gatherings, social distancing, international travel control measures, and contact tracing. Furthermore, evidence from the published literature appears to be largely congruent with the studied perceptions of national subject matter experts from European countries who were actively engaged in research, policy and policy advice during the pandemic. This knowledge is very important for public health decision-makers to be better prepared for the next pandemic.

## Data availability statement

The raw data supporting the conclusions of this article will be made available by the authors, without undue reservation.

## Author contributions

MSP, LL, and CP conceptualized the manuscript. MSP designed the search string. MSP, LL, MM, and MP have screened and extracted data. MSP and CP designed the modified Delphi surveys. MSP and MP wrote the draft of the manuscript. All authors revised the manuscript and approved its final version.

## Funding

This project has received funding from the European Union’s Horizon 2020 research and innovation program under grant agreement No. 101018317. The present publication was also funded by Fundação Ciência e Tecnologia, IP national support through CHRC (UIDP/04923/2020).

## References

[1] World Health Organization. (2023). Public Health and Social Measures. Available at: https://www.who.int/emergencies/diseases/novel-coronavirus-2019/phsm (Accessed May 17, 2023)

[2] Cowling BJ Ali ST Ng TWY Tsang TK Li JCM Fong MW. Impact assessment of non-pharmaceutical interventions against coronavirus disease 2019 and influenza in Hong Kong: an observational study. Lancet Public Health. (2020) 5:e279–88. doi: 10.1016/S2468-2667(20)30090-6 32311320 PMC7164922

[3] Aristodemou K Buchhass L Claringbould D. The COVID-19 crisis in the EU: the resilience of healthcare systems, government responses and their socio-economic effects. Eurasian Econ Rev. (2021) 11:251–81. doi: 10.1007/s40822-020-00162-1

[4] Ivanković D Barbazza E Bos V Fernandes ÓB Gilmore KJ Jansen T. Features constituting actionable COVID-19 dashboards: descriptive assessment and expert appraisal of 158 public web-based COVID-19 dashboards. J Med Internet Res. (2021) 23:e25682. doi: 10.2196/25682 33577467 PMC7906125

[5] Khodaveisi T Dehdarirad H Bouraghi H Mohammadpour A Sajadi F Hosseiniravandi M. Characteristics and specifications of dashboards developed for the COVID-19 pandemic: a scoping review. J Public Health. (2023):1–22. doi: 10.1007/S10389-023-01838-Z/FIGURES/3 PMC9894516 36747505

[6] European Union. (2020). Re-open EU. Available at: https://reopen.europa.eu/en (Accessed July 11, 2022)

[7] European Health Information Portal. (2021). Health Information Portal | European Health Information Portal. Available at: https://www.healthinformationportal.eu/health-information-portal (Accessed August 10, 2022)

[8] Population Health Information Research Infrastructure. (2021). The PHIRI project | phiri.eu. Available at: https://www.phiri.eu/ (Accessed August 10, 2022)

[9] Hale Thomas Angrist Noam Cameron-Blake Emily Hallas Laura Kira Beatriz Majumdar Saptarshi. (2020). Coronavirus government response tracker | Blavatnik School of Government. Oxford COVID-19 government response tracker. Available at: https://www.bsg.ox.ac.uk/research/research-projects/coronavirus-government-response-tracker (Accessed January 11, 2021) 10.1038/s41562-021-01079-8 33686204

[10] University of Oxford. (2021). World’s first COVID-19 government response tracker launched today | Blavatnik School of Government. Available at: https://www.bsg.ox.ac.uk/research/research-projects/covid-19-government-response-tracker (Accessed October 4, 2021)

[11] GitHub. (2023). OxCGRT/covid-policy-tracker…GitHub. Available at: https://github.com/OxCGRT/covid-policy-tracker/blob/master/documentation/codebook.md (Accessed May 17, 2023)

[12] Walsh S Chowdhury A Braithwaite V Russell S Birch JM Ward JL. Do school closures and school reopenings affect community transmission of COVID-19? A systematic review of observational studies. BMJ Open. (2021) 11:e053371. doi: 10.1136/BMJOPEN-2021-053371 PMC8375447 34404718

[13] Viner R Waddington C Mytton O Booy R Cruz J Ward J. Transmission of SARS-CoV-2 by children and young people in households and schools: a meta-analysis of population-based and contact-tracing studies. J Infect. (2022) 84:361–82. doi: 10.1016/J.JINF.2021.12.026 34953911 PMC8694793

[14] Iezadi S Gholipour K Azami-Aghdash S Ghiasi A Rezapour A Pourasghari H. Effectiveness of non-pharmaceutical public health interventions against COVID-19: a systematic review and meta-analysis. PLoS One. (2021) 16:e0260371. doi: 10.1371/JOURNAL.PONE.0260371 34813628 PMC8610259

[15] Irfan O Li J Tang K Wang Z Bhutta ZA. Risk of infection and transmission of SARS-CoV-2 among children and adolescents in households, communities and educational settings: a systematic review and meta-analysis. J Glob Health. (2021) 11:1–15. doi: 10.7189/JOGH.11.05013 PMC8285769 34326997

[16] Krishnaratne S Littlecott H Sell K Burns J Rabe JE Stratil JM. Measures implemented in the school setting to contain the COVID-19 pandemic. Cochrane Database Syst Rev. (2022) 1:CD015029. doi: 10.1002/14651858.CD015029 35037252 PMC8762709

[17] Krishnaratne S Pfadenhauer LM Coenen M Geffert K Jung-Sievers C Klinger C. Measures implemented in the school setting to contain the COVID-19 pandemic: a rapid scoping review. Cochrane Database Syst Rev. (2020) 12:CD013812. doi: 10.1002/14651858.CD013812 33331665 PMC9206727

[18] Viner RM Russell SJ Croker H Packer J Ward J Stansfield C. School closure and management practices during coronavirus outbreaks including COVID-19: a rapid systematic review. Lancet Child Adolesc Health. (2020) 4:397–404. doi: 10.1016/S2352-4642(20)30095-X 32272089 PMC7270629

[19] Viner RM Mytton OT Bonell C Melendez-Torres GJ Ward J Hudson L. Susceptibility to SARS-CoV-2 infection among children and adolescents compared with adults: a systematic review and meta-analysis. JAMA Pediatr. (2021) 175:143–56. doi: 10.1001/JAMAPEDIATRICS.2020.4573 32975552 PMC7519436

[20] Rizvi RF Craig KJT Hekmat R Reyes F South B Rosario B. Effectiveness of non-pharmaceutical interventions related to social distancing on respiratory viral infectious disease outcomes: a rapid evidence-based review and meta-analysis. SAGE Open Med. (2021) 9:205031212110229. doi: 10.1177/20503121211022973 PMC8188982 34164126

[21] Sun KS Lau TSM Yeoh EK Chung VCH Leung YS Yam CHK. Effectiveness of different types and levels of social distancing measures: a scoping review of global evidence from earlier stage of COVID-19 pandemic. BMJ Open. (2022) 12:e053938. doi: 10.1136/BMJOPEN-2021-053938 PMC9002256 35410924

[22] Ayouni I Maatoug J Dhouib W Zammit N Ben FS Ghammam R. Effective public health measures to mitigate the spread of COVID-19: a systematic review. BMC Public Health. (2021) 21:1015. doi: 10.1186/S12889-021-11111-1 34051769 PMC8164261

[23] Mendez-Brito A El Bcheraoui C Pozo-Martin F. Systematic review of empirical studies comparing the effectiveness of non-pharmaceutical interventions against COVID-19. J Infect. (2021) 83:281–93. doi: 10.1016/J.JINF.2021.06.018 34161818 PMC8214911

[24] Talic S Shah S Wild H Gasevic D Maharaj A Ademi Z. Effectiveness of public health measures in reducing the incidence of COVID-19, SARS-CoV-2 transmission, and COVID-19 mortality: systematic review and meta-analysis. BMJ. (2021) 375:e068302. doi: 10.1136/BMJ-2021-068302 34789505 PMC9423125

[25] Walsh KA Tyner B Broderick N Harrington P O’Neill M Fawsitt CG. Effectiveness of public health measures to prevent the transmission of SARS-CoV-2 at mass gatherings: a rapid review. Rev Med Virol. (2022) 32:2285. doi: 10.1002/RMV.2285 34390056

[26] Chu DK Akl EA Duda S Solo K Yaacoub S Schünemann HJ. Physical distancing, face masks, and eye protection to prevent person-to-person transmission of SARS-CoV-2 and COVID-19: a systematic review and meta-analysis. Lancet. (2020) 395:1973–87. doi: 10.1016/S0140-6736(20)31142-9, PMID: 32497510 PMC7263814

[27] Shah K Saxena D Mavalankar D. Secondary attack rate of COVID-19 in household contacts: a systematic review. QJM. (2020) 113:841–50. doi: 10.1093/QJMED/HCAA232 32726452 PMC7454929

[28] Patiño-Lugo DF Vélez M Salazar PV Vera-Giraldo CY Vélez V Marín IC. Non-pharmaceutical interventions for containment, mitigation and suppression of COVID-19 infection. Colomb Med. (2020) 51:1–25. doi: 10.25100/CM.V51I2.4266 PMC7518730 33012884

[29] Girum T Lentiro K Geremew M Migora B Shewamare S Shimbre MS. Optimal strategies for COVID-19 prevention from global evidence achieved through social distancing, stay at home, travel restriction and lockdown: a systematic review. Arch Public Health. (2021) 79:1–18. doi: 10.1186/S13690-021-00663-8/TABLES/3 34419145 PMC8380106

[30] Khosravizadeh O Ahadinezhad B Maleki A Najafpour Z Golmohammadi R. Social distance capacity to control the COVID-19 pandemic: a systematic review on time series analysis. Int J Risk Saf Med. (2022) 33:5–22. doi: 10.3233/JRS-210037 34719440

[31] Yuen E Fried J Salvador C Gudis DA Schlosser RJ Nguyen SA. Nonpharmacological interventions to reduce respiratory viral transmission: an evidence-based review with recommendations. Rhinology. (2021) 59:114–32. doi: 10.4193/RHIN20.563 33760909

[32] Abboah-Offei M Salifu Y Adewale B Bayuo J Ofosu-Poku R Opare-Lokko EBA. A rapid review of the use of face mask in preventing the spread of COVID-19. Int J Nurs Stud Adv. (2021) 3:100013. doi: 10.1016/J.IJNSA.2020.100013 33313575 PMC7718106

[33] Tran TQ Mostafa EM Tawfik GM Soliman M Mahabir S Mahabir R. Efficacy of face masks against respiratory infectious diseases: a systematic review and network analysis of randomized-controlled trials. J Breath Res. (2021) 15:ac1ea5. doi: 10.1088/1752-7163/AC1EA5 34407516

[34] Ford N Holmer HK Chou R Villeneuve PJ Baller A van Kerkhove M. Mask use in community settings in the context of COVID-19: a systematic review of ecological data. EClinicalMedicine. (2021) 38:101024. doi: 10.1016/j.eclinm.2021.101024 34308320 PMC8287197

[35] Girum T Lentiro K Geremew M Migora B Shewamare S. Global strategies and effectiveness for COVID-19 prevention through contact tracing, screening, quarantine, and isolation: a systematic review. Trop Med Health. (2020) 48:91. doi: 10.1186/S41182-020-00285-W 33292755 PMC7680824

[36] Chung SC Marlow S Tobias N Alogna A Alogna I You SL. Lessons from countries implementing find, test, trace, isolation and support policies in the rapid response of the COVID-19 pandemic: a systematic review. BMJ Open. (2021) 11:e047832. doi: 10.1136/BMJOPEN-2020-047832 PMC8251680 34187854

[37] Bou-Karroum L Khabsa J Jabbour M Hilal N Haidar Z Abi Khalil P. Public health effects of travel-related policies on the COVID-19 pandemic: a mixed-methods systematic review. J Infect. (2021) 83:413–23. doi: 10.1016/J.JINF.2021.07.017 34314737 PMC8310423

[38] Nussbaumer-Streit B Mayr V Dobrescu AI Chapman A Persad E Klerings I. Quarantine alone or in combination with other public health measures to control COVID-19: a rapid review. Cochrane Database Syst Rev. (2020) 2020:CD013574. doi: 10.1002/14651858.CD013574 PMC8133397 33959956

[39] Caristia S Ferranti M Skrami E Raffetti E Pierannunzio D Palladino R. Effect of national and local lockdowns on the control of COVID-19 pandemic: a rapid review. Epidemiol Prev. (2020) 44:60–8. doi: 10.19191/EP20.5-6.S2.104 33412795

[40] Grépin KA Ho TL Liu Z Marion S Piper J Worsnop CZ. Evidence of the effectiveness of travel-related measures during the early phase of the COVID-19 pandemic: a rapid systematic review. BMJ Glob Health. (2021) 6:e004537. doi: 10.1136/BMJGH-2020-004537 PMC7969755 33722793

[41] Viswanathan M Kahwati L Jahn B Giger K Dobrescu AI Hill C. Universal screening for SARS-CoV-2 infection: a rapid review. Cochrane Database Syst Rev. (2020) 2020:CD013718. doi: 10.1002/14651858.CD013718 PMC8453488 33502003

[42] Mbwogge M. Mass testing with contact tracing compared to test and trace for the effective suppression of COVID-19 in the United Kingdom: systematic review. JMIRx Med. (2021) 2:e27254. doi: 10.2196/27254 33857269 PMC8045129

[43] Mazza C Girardi D Gentile L Gaeta M Signorelli C Odone A. Public health effectiveness of digital contact tracing in the COVID-19 pandemic: a systematic review of available data. Acta Biomed. (2021) 92:e2021439. doi: 10.23750/ABM.V92IS6.12237 34889315 PMC8851023

[44] Jenniskens K Bootsma MCJ Damen JAAG Oerbekke MS Vernooij RWM Spijker R. Effectiveness of contact tracing apps for SARS-CoV-2: a rapid systematic review. BMJ Open. (2021) 11:e050519. doi: 10.1136/BMJOPEN-2021-050519 PMC8277487 34253676

[45] Craig KJT Rizvi R Willis VC Kassler WJ Jackson GP. Effectiveness of contact tracing for viral disease mitigation and suppression: evidence-based review. JMIR Public Health Surveill. (2021) 7:e32468. doi: 10.2196/32468 34612841 PMC8496751

[46] Hossain AD Jarolimova J Elnaiem A Huang CX Richterman A Ivers LC. Effectiveness of contact tracing in the control of infectious diseases: a systematic review. Lancet Public Health. (2022) 7:e259–73. doi: 10.1016/S2468-2667(22)00001-9 35180434 PMC8847088

[47] Becker LA Oxman AD. Overviews of reviews In: Cochrane Handbook for Systematic Reviews of Interventions: Cochrane Book Series. eds. Julian PT Higgins and Sally Green Hoboken, NJ: Wiley (2008). 607–31.

[48] Page MJ McKenzie JE Bossuyt PM Boutron I Hoffmann TC Mulrow CD. The PRISMA 2020 statement: an updated guideline for reporting systematic reviews. BMJ. (2021) 372:n71. doi: 10.1136/bmj.n71 33782057 PMC8005924

[49] Babineau J. Product review: Covidence (systematic review software). J Canad Health Lib Assoc. (2014) 35:68. doi: 10.5596/c14-016

[50] Whiting P Savović J Higgins JPT Caldwell DM Reeves BC Shea B. ROBIS: a new tool to assess risk of bias in systematic reviews was developed. J Clin Epidemiol. (2016) 69:225–34. doi: 10.1016/j.jclinepi.2015.06.005 26092286 PMC4687950

[51] Niederberger M Spranger J. Delphi technique in health sciences: a map. Front Public Health. (2020) 8:457. doi: 10.3389/FPUBH.2020.00457/BIBTEX 33072683 PMC7536299

