# Supplementary material for: Impacts of public health and social measures on COVID-19 in Europe: a review and modified Delphi technique
Source: Front Public Health. 2023 Aug 31;11:1226922. doi: 10.3389/fpubh.2023.1226922 (PMC10501783; doi:10.3389/fpubh.2023.1226922)
Supplement: Supplementary file 1 [file Table_1.DOCX]

Supplementary Material

Health policies and public health and social measures impact measures on COVID-19 in Europe: a review and modified Delphi technique

Marília Silva Paulo^1,2*^, Mariana Peyroteo^1,3^, Mélanie R. Maia^3,4^, Cara Pries^5^, Claudia Habl^5^, Luís Velez Lapão^1,3,4,6^

*** Correspondence:** Marília Silva Paulo, [marilia.paulo@nms.unl.pt](mailto:marilia.paulo@nms.unl.pt)

# Informed consent and modified Delphi data collection tools

## Informed consent

Dear all, we are inviting you to participate in a Modified Delphi Panel study aiming to define the three PHSMs that you consider to have most impacted the epidemiological curve of COVID-19 over the last two years or what is considered its four waves.

As you know, since the beginning of the COVID-19 pandemic in early 2020, all the countries in the world have implemented several public health and social measures (PHSMs) to try to contain the spread of the SARS-CoV-2 virus.

We have designed a survey to be distributed to all countries among the PHIRI project members. We have selected the majority of our indicators from the Oxford COVID-19 Government Response Tracker (OxCGRT) which was developed by the Blavatnik School of Government, University of Oxford. During the selection of the indicators, the research team felt the need to include four additional indicators based on the literature search and the topics presented at the REF meetings based on countries' requests. In the first stage of the survey, the country member will select three PHSMs as those considered most important to control or decrease the epidemiological curve of COVID-19 during each wave of the pandemic. After receiving the answers, we will analyze them and create another survey where we will ask the country member to rate these PHSMs by order of importance. As a result, we will have an agreement on the most impactful measures taken.

Your participation in this survey is voluntary and no benefits will be received. We also ensure that your participation is totally anonymous as no identifiable information will be asked.

## Questions

1. Which country are you from?
2. Please select from the list below the THREE public health and social measures that most impacted the epidemiological curve of COVID-19 during the first wave in your country:

Closure of schools/kindergartens

Workplace closure

Closure of non-essential shops, gastronomy, and cultural events

Access restrictions to shops, gastronomy, and cultural events

Cancellation of public events

Restriction on public gatherings

Social distancing

Hygiene measures

Face coverings (all types)

Voluntary quarantine by contacts

Case isolation at home

Public transports closures

Stay-at-home campaign

Restrictions international movement

International travel control

Testing policy (anyone with symptoms)

Contact tracing

Vaccination policy (all vulnerable groups)

Public information campaigns

1. Please select from the list below the THREE public health and social measures that most impacted the epidemiological curve of COVID-19 during the second wave in your country:

- Same options as question number two.

1. Please select from the list below the THREE public health and social measures that most impacted the epidemiological curve of COVID-19 during the third wave in your country:

- Same options as question number two.

1. Please select from the list below the THREE public health and social measures that most impacted the epidemiological curve of COVID-19 during the fourth wave in your country:

- Same options as question number two.

# Reason for excluded studies during data extraction

Supplementary Table 1: Reason for excluded studies during data extraction

| Study ID | Reason for exclusion |
| --- | --- |
| Akinbi 2021 | Does not look into the impact of the epidemiological curve of COVID-19 |
| Alvi 2020 | Study design: Narrative Review |
| Anwar 2020 | Study design: Narrative Review – no methods reported |
| Ayenigbara 2020 | Study design: Narrative Review – no methods reported |
| AnthonyJnr 2021 | Digital and Technological Features |
| Abboah-Offei 2021 | Does not look into the impact of the epidemiological curve of COVID-19 |
| Braithwaite 2020 | Study design: Modelling study |
| Burns 2021 | Study design: Modelling study |
| Busa 2021 | Study design: Narrative Review |
| Chetty 2020 | Study design: Modelling study |
| ElBcheraoui 2021 | Wrong (infectious) diseases |
| Halperin 2021 | Study design: Narrative Review – no methods reported |
| JuJTJ 2021 | No methods reported |
| López-Bueno 2021 | Does not look into the impact of the epidemiological curve of COVID-19 |
| Misra 2022 | Does not look into the impact of the epidemiological curve of COVID-19 |
| Mbunge 2020 | Digital and Technological Features + Does not look into the impact of the epidemiological curve of COVID-19 |
| Nanda 2021 | Does not split results per NPI separately |
| Nenna 2021 | Study design: Narrative Review without methods details |
| Odusanya 2020 | Study design: Narrative Review without methods details |
| Ogbuoji 2021 | Does not look into the impact of the epidemiological curve of COVID-19 |
| Perra 2021 | Study design: Review – author chose the papers included based on its own criteria |
| Polisena 2021 | Does not look into the impact of the epidemiological curve of COVID-19 – identification of 722 PH measures implemented in Canada |
| Rahman 2022 | Does not look into the impact of the epidemiological curve of COVID-19 |
| Regmi 2021 | Does not look into the impact of the epidemiological curve of COVID-19 – factors associated with implementation of NPIs |
| Tucho 2021 | Study design: Narrative Review – no methods reported |
| Rooney 2021 | Studies from other infectious diseases without a direct impact on the epidemiological curve of COVID-19 |
| Rowan 2021 | Study design: Narrative Review without methods details |
| Seale 2020 | Does not look into the impact of the epidemiological curve of COVID-19 – factors associated with compliance of NPIs |
| Tirupathi 2020 | Study design: Narrative Review – no methods reported |
| Tully 2021 | Does not look into the impact of the epidemiological curve of COVID-19 – effect of NPIs on Google mobility |

# Risk of bias assessment

Supplementary Figure 1: Risk of bias assessment of the included studies.
